# Automated quantity take-off in a Norwegian road project

**DOI:** 10.1038/s41598-023-50486-6

**Published:** 2024-01-03

**Authors:** David Fürstenberg, Eilif Hjelseth, Ole Jonny Klakegg, Jardar Lohne, Ola Lædre

**Affiliations:** 1 Department of Civil and Environmental Engineering, Norwegian University of Science and Technology, Høgskoleringen 7a, 7491 Trondheim, Norway; 2 COWI AS, Bergen, Norway

**Keywords:** Engineering, Civil engineering

## Abstract

Automated quantity take-off from digital models in road projects has many benefits. In real project settings, however, it seems rarely to take place. This paper investigates automated quantity take-off in a real-life road project and the experiences from this automation. A literature and a document study were performed. 48 domain models serialized in IFC 2 × 3 were then analyzed before 10 project members were interviewed. The quantity take-off was automated to harvest classified quantities to a specification of work. The automated quantity take-off was reproducible at revisions for 40% of the cost items. However, the transferability of the procedures to other use cases than the specification of work is questionable because of the applied cost breakdown structure. The findings suggest three practical improvements for automated quantity take-off in road projects. The three include (1) using unambiguous classes assembled in an ontology, (2) avoiding hard coding of cost breakdown structures in authoring tools, and (3) implementing the Level of Information Need (LOIN) to improve reusability across project phases and use cases. The quantity take-off and experiences are assessed from the designer's perspective. The client’s and contractor’s perspectives were out of this paper’s scope.

## Introduction

Quantity take-off is among the most important recurring processes for designers^1,2^. It is the basis for many subsequent activities, such as cost estimation, calculation of greenhouse gases, assessment of environmental impacts, or scheduling. The benefits of quantity take-off from digital models, like reducing human error or saving time, are well described in the literature^3–8^. The focus of the existing research on model-based quantity take-off is mainly on buildings and less on infrastructure. One reason might be the early introduction of the open Industry Foundation Classes (IFC) which are based on a breakdown structure adapted to buildings and not to infrastructures such as roads.

During the last decade, model-based design has become a client demand for public buildings and infrastructure projects in many countries. While digital models can be used as a supplement to traditional drawings, Norwegian public infrastructure clients enforce a "model-only" policy^9^. Huge infrastructure projects are designed, approved, and built completely without drawings. Automated quantity take-off should be possible in this "model-only" environment, but is yet to become state-of-the-art. The degree to which real-life experiences correspond to the theoretical potential has not so far been properly examined for road projects. Simply put, automated quantity take-off from digital models in road projects has many benefits. In real project settings, however, it seems rarely to take place. Consequently, the practical obstacles to automated quantity take-off in road projects are rarely documented.

This paper investigates automated quantity take-off in a road project. More precisely the overall objective of the analysis is to investigate contemporary approaches to the automation of quantity take-off. The following research questions were addressed:

### RQ1

How is quantity take-off automated in the investigated case?

### RQ2

What are the experiences with automated quantity take-off?

The experiences are assessed from the designer's perspective using an action research approach that enabled an in-depth understanding of how quantity take-off was automated. The client's and contractor's perspectives were out of this paper’s scope.

## Theoretical background

### Challenges with model-based quantity take-off

Model-based quantity take-off is challenging. Inconsistent and/or omitted information are typical challenges for model-based quantity take-off^10–14^. Wu et al.^15^ report on data exchange issues between different stakeholders. Liu et al.^16^ report on the cumbersome manual work of aggregating quantities and extracting implicit information from models to complete the quantity take-off. Monteiro and Martins^5^ report on inaccurate modelled objects as a challenge for model-based quantity take-off and suggest modeling guidelines. More recently, Liu et al.^17^ and Khosakitchalert et al.^18^ presented novel modeling methods to raise the geometric accuracy of modelled objects.

In short, challenges in model-based quantity take-off for buildings and infrastructures mentioned by literature are inconsistent information, the need for human expert knowledge, geometric inaccuracy, and data exchange issues. These challenges might hinder the standardization (and thereby the reuse) of a model-based quantity take-off.

### Automation of model-based quantity take-off

Several studies report on how model-based quantity take-off can be automated. Reusing classified model entities seems to be the most common way^19,20^. One way to classify is to use proprietary model classes^21–23^ or open model classes based on the Industry Foundation Classes (IFC). Examples of the latter are Isatto^24^ and Choi et al.^25^. Another way is to code model entities according to a common classification system, either directly within an authoring tool^26–29^ or within a specialized cost management tool^30–32^. Yet another way to code model entities is by applying standardized concepts through semantic web technology, known as ontologies. Lee et al.^33^ created ontologies for work items and work conditions. Liu et al.^16^ developed a product ontology for light-framing building construction. Several authors presented ontologies based on existing classification systems. Ma et al.^34^ created an ontology based on a Chinese cost classification system and Fürstenberg et al.^35^ presented a prototype based on a Norwegian cost classification system for infrastructure projects. Abanda et al.^36^ and Xu et al.^37^ developed an ontology for the New Rules of Measurements used in the UK. Niknam and Karshenas^38^ created an ontology for the Uniform II classification system.

In short, several technical approaches exist for automating the quantity take-off and all involve a classification. However, based on the first author’s 15 years of professional experience from an international design consulting company, it seems that automated quantity take-off is still not state-of-the-art, not even in Norwegian “model-only” road projects.

### Standards relevant for automated quantity take-off in road projects

Classification systems are used to break down construction projects into work packages, cost items, or objects that aim to represent any part of the perceivable or conceivable world^39^. In construction, these objects can be either an element (if approached from a result vision) or a product (if the approach is resources). The ISO 12006-2 standard unites both approaches. Several international classification systems are based on this standard, e.g., Uniclass 2015 from the UK, Omniclass from the US, or the European CCI. Extending the ISO 12006-2, the ISO 19650 series on information requirements were developed. Related to the ISO 19650 series is the Level of Information Need—LOIN—(EN 17412-1:2020) which defines "the extent and granularity of information to be exchanged".

However, the Norwegian Public Roads Administration (NPRA) have had a different approach preferring national employer-specific standards over ISO standards^40^. In the context of the investigated case, especially two of these employer-specific standards are relevant. The first is a standard on information requirements published in the handbook V770^41^. The second is a standard catalog of work tasks for building roads mandatory in all NPRA projects delivered as design-bid-build. The work tasks are split into eight main classes published in the handbooks R761^42^ and R762^43^. These main classes roughly correspond to the upper level of a breakdown structure defined by the V770, so-called discipline models representing subdomain models.

For the lower level of the breakdown structure, the V770 prescribes a system based on all R761/R762 work tasks. R761 contains the main classes 1–7 which describe all work tasks necessary in a road project except bridge-related tasks. R762 contains main class 8 which describes only bridge-related tasks. All classes are open for user-defined extensions using additional non-standard codes. A specification text in these handbooks is called a (work) process. A process consists of a number, a title, a specific unit, and in most cases detailed requirements for the scope, materials, tolerances, and quantity take-off. An object-type library does not yet exist for all road assets but is under development.

Other breakdown structures can be used as well, both open and proprietary. The Industry Foundation Classes (IFC/ISO 16739) represent the most common open breakdown structure. At the time of investigation, two IFC versions are covered by ISO 16739, namely IFC 2 × 3 and IFC 4 add 2. However, since road-specific classes are not supported in either of these versions, IFC is mainly used as an open data container in road projects. Road-specific classes will be supported from IFC version 4 × 3 which is under ISO evaluation. Authoring tools like Novapoint, Revit, or Tekla use proprietary breakdown structures for their model classes. While the breakdown structure of Revit and Tekla was intended for an international market, Novapoint's breakdown structure was initially created for the Norwegian market based on Norwegian standards.

### The dominating tools for automated quantity take-off in Norwegian road projects

ISY Beskrivelse—"beskrivelse" means description/specification of work in English—is one of two tools for creating combined bills of quantities and specification of work in the Norwegian market. The usage of one of these two tools has become a domain standard. Both tools have a built-in database containing all information from the catalog of standard work tasks (R761/R762). Both tools can import classified quantities from IFC files and merge them automatically with standard specification texts. For easier reading, we will from here on refer to ISY Beskrivelse as ISY Description.

### Knowledge gap

Automated quantity take-off is easier for building projects than for road projects, but both types of projects can benefit. Even though the road-specific obstacles hinder standardization and thereby automation of quantity take-off, several studies have shown feasible technical solutions to the challenges based on different ways of classifying model entities. However, these studies focused on buildings and/or single-domain issues restricted to a theoretical context. Little priority has so far been given to the practical applicability and reusability of automated quantity take-off in multi-domain road projects within a highly digitally developed environment. Moreover, based on the first author’s 15 years of professional experience from an international design consulting company, it seems that automated quantity take-off is still not state-of-the-art, not even in “model-only” Norwegian road projects. Therefore, the analysis presented here enriches the theoretical understanding with novel real-life experiences from a Norwegian "model only" road project.

## Methodology

### Research design

Details are important when investigating digitalization. To understand how quantity take-off was automated in the investigated case and document the experiences with automated quantity take-off, in-depth experience with the everyday workings of the road project proved necessary. The first author was the BIM manager in the initial phase of the studied case project. Thereafter, he supported the succeeding BIM manager during the design phase while establishing an automated quantity take-off. Both the first author and the succeeding BIM Manager were employed by the international design consulting company COWI. Since the first author had work experience from the selected case, he got full access to project documentation and an understanding of how the quantity take-off was automated.

Constructivist researchers maintain that researchers perceive reality subjectively and are influenced by their social setting^44^. The research questions were investigated from the designers’ perspective, and the social setting in the design team may have influenced the first author’s perception of reality.

While Somekh^45^ stressed that action researchers might interpret results in the light of prior knowledge, Coughlan and Coghlan^46^ recommended action research to collect data from real-life projects. An action research approach was chosen because it was a study of hands-on experiences in a real-life project.

### Case description—a road project with automated quantity take-off

The case examined was a road project in Western Norway, consisting of 7 km of single-lane highway and 3 km of secondary roads. The highway and roads included two roundabouts, two two-level crossings, 25 constructions, landscaping, and associated systems for water and sewer and cabling. In 2021, the total construction costs were estimated to be approximately 10 000 000€. The domains road, construction, water and sewer, and electrical were responsible for the main quantities in the project. The landscaping domain produced additional quantities. The delivery method was design-bid-build and the studied project phase was detailed designing as defined in the Norwegian phase norm “Next step”^47^. The quantity take-off was automated to harvest classified quantities to a specification of work following the Norwegian R761/R762 classes. Producing a specification of work following the R761/R762 classes is mandatory in NPRA projects delivered as design-bid-build.

At the time of investigation, the models were prepared for final delivery with a model maturity index (MMI) of 390. The Norwegian MMI defines several maturity levels. It has similarities to the Level of Development (LoD) but describes both the level of geometry and the level of information. In the investigated case a project-specific index of 390 indicated that the model was ready for tendering but not yet approved by the contractor.

The authors chose this case because the NPRA requested a model-only design and was willing to spend extra funds on automating quantity take-off. COWI was responsible for the road design. Only standard software tools were used for design, even though COWI has automation of design processes at the core of its business strategy.

3.3 Research methods—literature review, document study, observations, and interviews.

The initial literature review followed the steps described by Blumberg et al.^48^: (1) build an information pool, (2) apply filters to reduce pool size, (3) rough assessment of sources to further reduce pool size, (4) analyze literature in the pool and (5) refine filters or stop the search.

For the case study, the prescriptions outlined by^49^ were followed. Documentation, observation, and focused interviews were used as sources of evidence. The client's information requirements (V770), the standard catalog of work tasks (R761/R762), a combined bill of quantities and specification of work, and several BIM-related documents were studied in detail. BIM-related documents included the project's BIM execution plan, guidelines, and templates for assigning information to the models, and detailed descriptions of workflows. In addition, the case project’s 48 domain models serialized in IFC 2 × 3, were assembled into a federated Solibri model. From this federated model, specific information relevant to quantity take-off was exported to an Excel spreadsheet. Parts of the documents studied were written entirely or in part by the first author of the paper. The documents were chosen because they contained information related to quantity take-off in the project. According to Bowen^50^, a document study is an efficient way to supplement data gathered from interviews.

The first author attended weekly project meetings as an action researcher while working as a BIM manager. Their average duration was approximately 1.5 h. The project manager, the BIM manager, and the discipline leaders participated in these meetings. The number of participants varied between 5 and 7. There was no change of personnel during the design phase. Detailed meeting minutes were elaborated on by the project manager and made accessible to the first author. These meeting minutes stressed the preparation of automated quantity take-off. All meetings were digital, and the participation of the different actors varied largely—from being highly active to quite silent. The first author made observations during these weekly meetings and made a reflection note describing the relevant observations after each meeting.

The project manager, the BIM manager, four discipline BIM coordinators, and four discipline leaders were chosen as interview objects because they represented different perspectives. Focused interviews as described by Yin^49^ were carried out. The case-specific interviews lasted between 15 and 30 min and were summarized in a transcript. The ten interviews supplemented the data from the document study and the observations. The experiences from the automated quantity take-off were the red thread in the interviews.

### Data analysis

This study was approached according to a pre-understanding of the topic and analyzed the data with a hermeneutic-interpretive approach inspired by Ricoeur^51^. The goal of the analysis was to fill the knowledge gap, namely presenting practical examples from road projects that focus on transformed workflows to reap the benefits of digital ways of working. The unit of analysis was the reuse of the applied procedures within the case investigated—at each revision—and the transfer of these procedures to other projects.

### Limitations

This study reports on one Norwegian road project utilizing commercial state-of-the-art software available in the Norwegian market. ISY Description is mainly used in Norway, as it follows the standard Norwegian catalog of work tasks (R761/R762). The other tools represent internationally state-of-the-art (Civil 3d, Revit, Dynamo, Rhino, Grasshopper, Solibri) or are at least available in other markets (Novapoint, Tekla, SimpleBIM).

Presenting fresh data from a real-life “model only” project had priority over a broader and more general analysis of automated quantity take-off. The study presents hands-on experiences from a road project where digitalization was a client demand. The core of the enquiry was the designers' perspective on quantity take-off on the project level. Therefore, neither market directors nor higher-level managers from the designer were interviewed.

## Findings

This paper set out to investigate automated quantity take-off in a road project. To investigate contemporary approaches to the automation of quantity take-off, two research questions were addressed, namely (1) how quantity take-off is automated in the investigated case and (2) what the experiences with automated quantity take-off are.

### Automated quantity take-off in the investigated case

The above-mentioned domains—road, construction, water and sewer, electrical, and landscaping—created 48 domain models serialized in IFC 2 × 3, consisting of approximately 176.000 geometric entities. IFC 2 × 3 was used because COWI had established routines for delivering IFC 2 × 3. Introducing IFC 4 × 3 was not feasible at the time of investigation because it was not yet supported by all tools—e.g., Solibri. Included in these 48 domain models are seven domain-specific earthworks models and 41 subdomain models according to the previously mentioned V770 breakdown structure. These 41 models belonged to the subdomains road (three models), road signs (one model), bridges and constructions (19 models), construction details (seven models), water and sewer (two models), cabling systems (two models), lighting (one model), and landscaping (two models). Four of the 41 subdomain models did not contain any cost items.

The combined bill of quantities and specification of work consisted of 486 cost items. Of these 486 cost items, 291 − approximately 60% − were created manually and 195 − approximately 40% − were created automatically. 51 cost items were necessary to describe all work tasks related to preparations and maintenance. All of them were created manually. Table 1 gives an overview of the cost items' distribution across domains and their share of manual or automated creation.

**Table 1:** Cost items per domain represented in the case project.

Domain	Cost items (manually)		Cost items (automatically)		Total	
Preparations and maintenance	51	18%	0	0%	51	10%
Earthworks	59	20%	5	3%	64	13%
Water and sewer	18	6%	38	19%	56	12%
Electrical	37	13%	12	6%	49	10%
Road	102	35%	9	5%	111	23%
Landscape	12	4%	14	7%	26	5%
Construction	12	4%	117	60%	129	27%
Total	291	100%	195	100%	486	100%

The domains in the above table correspond—with some overlaps—to the R761/R762 main classes 1–8. Main class 1 contains all work tasks for preparation and maintenance during the construction phase, the unit of measurement is mainly lump sum. Main class 2 contains earthworks. Main class 3 contains work tasks on tunneling. In the investigated case no such work tasks existed. Main class 4 describes work tasks related to the water and sewer and electrical domains. Main classes 5 and 6 are work tasks mainly related to the road domain. Main class 7 contains work tasks related to the domains of road, construction, electrical, and landscaping. Main class 8 contains only construction-related work tasks. Table 2 shows the distribution of manually and automatically created cost items across the R761/R762 main classes and their corresponding domain.

**Table 2:** Cost items per main class according to Norwegian Public Roads Administration’s handbook R761 and R762 and their corresponding domain.

Main class	Domain	Cost items (manually)	Cost items (automatically)		Percentage automated per main class		Total	
1	Preparation and maintenance	51	18%	0	0%	0%	51	10%
2	Earthworks	59	20%	3	2%	5%	62	13%
3	Tunneling	0	0%	0	0%	0%	0	0%
4	Water and sewer/Electrical	39	13%	51	26%	57%	90	19%
5	Road	3	1%	6	3%	67%	9	2%
6	Road	14	5%	4	2%	22%	18	4%
7	Road, Construction, Electrical, Landscaping	115	40%	43	22%	27%	158	33%
8	Construction	10	3%	88	45%	90%	98	20%
Total		291	100%	195	100%		486	100%

**Figure 1:**
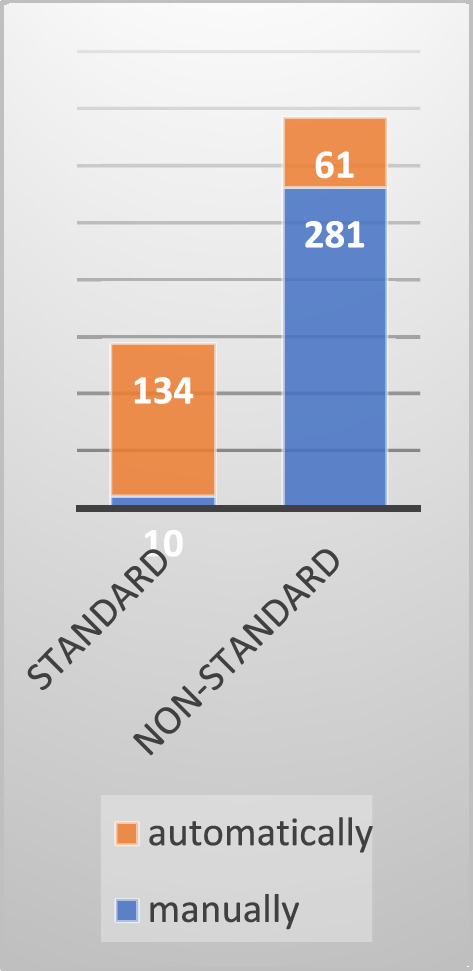
Distribution of cost items on standard and non-standard codes in the investigated case.

The client's standard catalog of work tasks is open for using non-standard codes. Figure 1 shows the distribution of all cost items—both manually and automatically created—across standard and non-standard codes.

**Figure 2:**
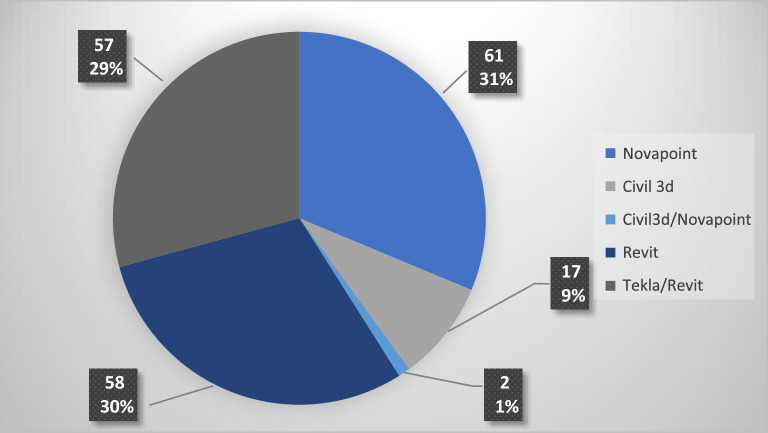
Authoring tool used for creating the automatically created cost items in the case project.

The domains used both databases (i.e., Novapoint) and file-based tools (i.e., Revit, Tekla, and Civil 3d) as authoring tools. The construction domain preferred Tekla and Revit in conjunction with their respective parametric extensions Rhino/Grasshopper and Dynamo. The domains road and water and sewer preferred Novapoint. The electrical domain used a combination of Novapoint and Civil 3D, while the landscaping domain preferred Civil 3D. Figure 2 shows the distribution of the authoring tools across the automatically created cost items.

**Figure 3:**
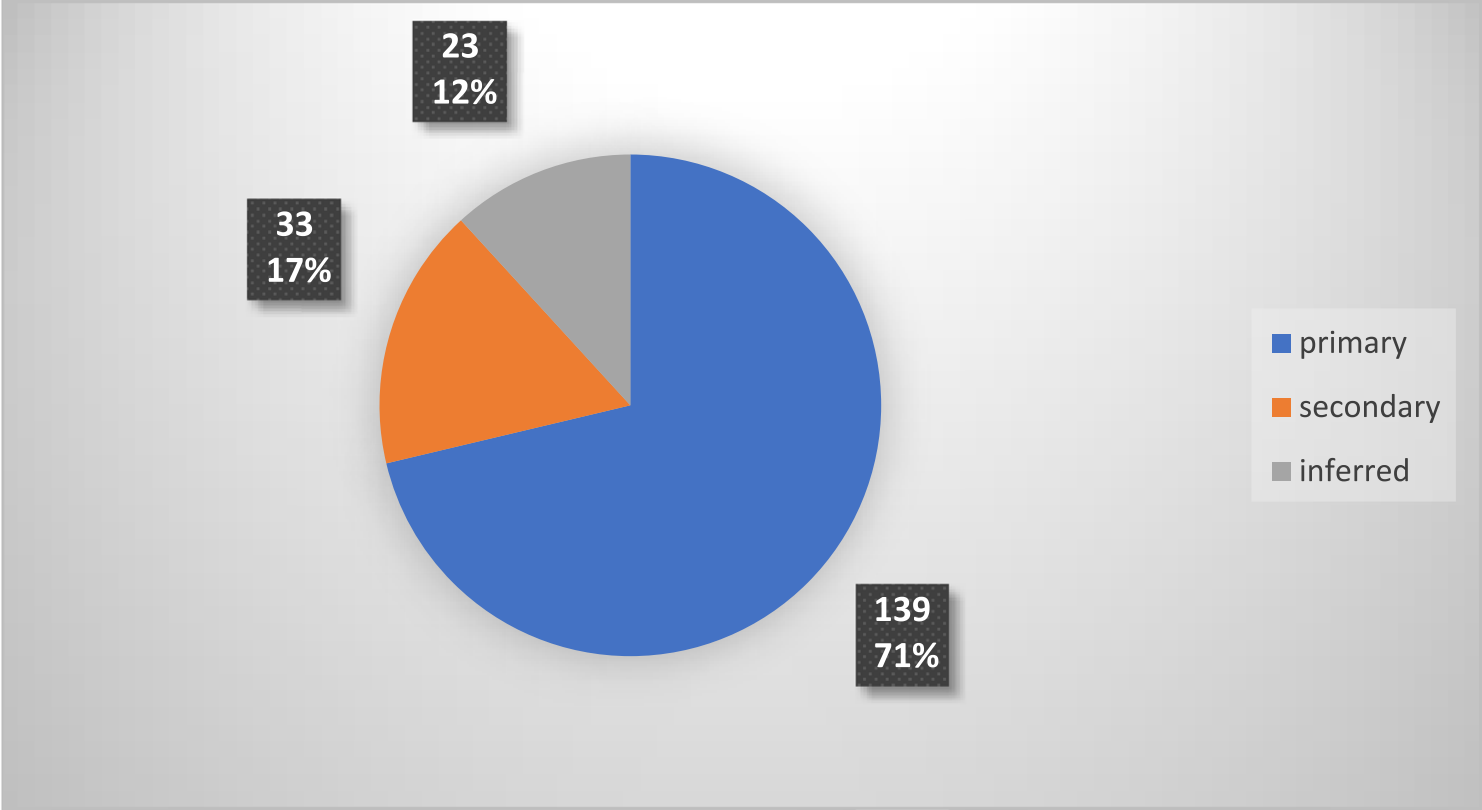
Types of measurement for the automatically created cost items.

The quantities of the automatically created cost items were categorized into three measurement types—primary, secondary, and inferred. Primary quantities were either counted per piece or measured directly in the models with the units m, m^2^, and m^3^. Secondary quantities were like primary quantities but created additional cost items with the same quantities as primary quantities. For example, the length of a water pipe—measured in meters—was a primary quantity while flushing, cleansing, disinfection, and pressure testing before the handover of the same pipe were secondary quantities—still measured in meters. Inferred quantities were either measured in lump sums or required formulas for calculation based on primary quantities. The latter was measured with the units kg, ton, meganewton, or hours. Some inferred cost items were indirectly created based on a percentage of a primary quantity. E.g., a concrete sole was calculated as a percentage of a natural stone wall sitting on top of it. Figure 3 shows the distribution of the automatically created cost items across the three measurement types.

### Automated quantity take-off in the investigated case

In the investigated case, quantity take-off was automated in six steps: (1) classification of proprietary model classes within authoring tools, (2) enriching the proprietary model classes with specific properties, (3) parsing the classified and enriched model classes to IFC 2 × 3, 4) rule-based checking for data consistency, (5) additional enriching outside the authoring tools, and (6) importing classified quantities to a specification tool. In short, a proprietary model breakdown structure was converted into a standardized cost breakdown structure readable by one specific tool.

**Figure 4:**
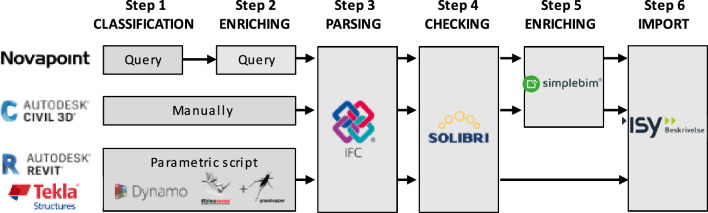
Automated quantity take-off in six steps for the investigated case.

Steps one, two, and four were only necessary initially. If only previously classified model classes were reused at revisions, only steps three, five, and six were necessary. Figure 4 illustrates the automated quantity take-off established in the investigated case.

The first step was to classify the proprietary model classes according to the R761/R762 classes by using one to four filters, depending on the authoring tool used. The first filter was based on V770 subdomain models and was applied in all authoring tools. Each subdomain model was represented either as a selection of a database (Novapoint) or as a separate file for the file-based authoring tools Tekla, Revit, and Civil 3d. In Novapoint the first filter was part of a query. Since Civil 3D was not an object-based tool, the classification was done manually for each geometric entity and no more filters were applied. In Revit and Tekla no more filters were applied, neither, and the classification was done by a parametric script in Dynamo or Rhino/Grasshopper.

**Figure 5:**
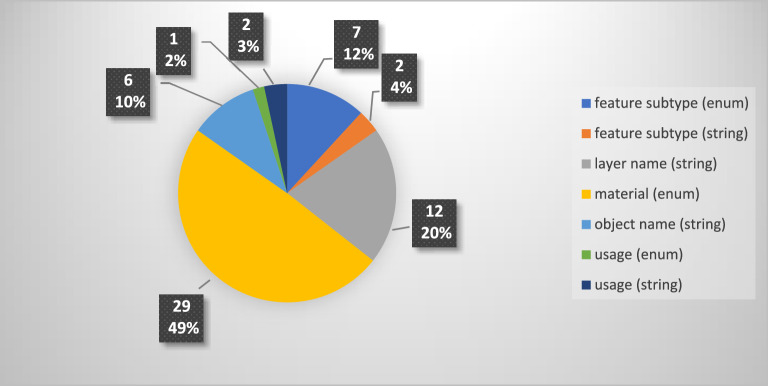
Distribution of the properties in seven types.

The second, third, and fourth filters were only applied in Novapoint. The second filter was on specific proprietary model classes and the third filter was on specific properties. These properties could be categorized into seven types. Enumerated values for materials were the predominant type (49%). Figure 5 illustrates the distribution of the seven property types. Four proprietary model classes—water pipeline, storm drain line, sewer pipeline, and conduit pipe—required a fourth filter based on the diameter or additional material.

The second step was to enrich the proprietary model classes—or geometric entities in Civil 3D—with specific properties required by the specification tool. These properties were the unit of measurement and internal localizers. In Civil 3d this step was done manually. In Revit and Tekla this step was incorporated in the parametric script used to classify the proprietary model classes mentioned in step 1.

**Figure 6:**
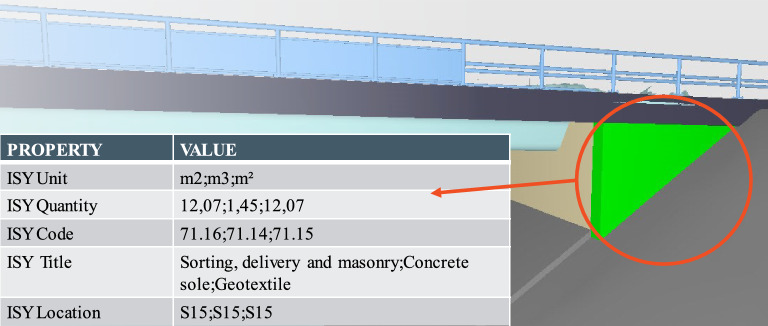
Custom property set in ISY Description containing quantities (12,07;1,45;12,07) for three cost items (71.16;71.14;71.15) necessary for building a natural stone retaining wall (highlighted in green).

The third step was to parse the classified and enriched proprietary model classes and serialize them in IFC 2 × 3. Information about the R761/R762 classes (code, title, unit of measurement), internal localizers, and the quantities was assembled into a specifically named property set. The property names and some of the values were in Norwegian. However, for the convenience of the reader, the names are translated into English. The R761/R762 classes were split and parsed into the properties ISY Code and ISY Title. The unit of measurement was parsed into the property ISY Unit. The internal localizers were parsed into ISY Location, and the quantity was parsed into ISY Quantity. Figure 6 illustrates this property set for a natural stone retaining wall.

The fourth step was a rule-based check for data consistency of the ISY Description property set. Solibri was used for this check.

In the fifth step, additional properties were added outside the authoring tools. This was partly done due to technical limitations of the authoring tools—Novapoint and Civil 3D—and partly done for process automation. Cost items with secondary or inferred quantities were created this way using Excel-based templates tailored for SimpleBIM. Secondary or inferred cost items were created by appending their properties to a primary quantity using a ";" as shown in Fig. 6. In Revit and Tekla step 5—in the meaning of adding additional properties—was incorporated in the parametric script mentioned for step 1. SimpleBIM was not used for IFC files parsed from Revit or Tekla. The number of secondary or inferred cost items varied per subdomain model, ranging from 1 to 17. Only models from the construction domain—using Revit or Tekla—had more than four secondary or inferred cost items. Table 3 gives an overview of subdomain models and the number of R761/R762 classes per model class.

**Table 3:** The number of R761/R762 classes per model class per subdomain model.

Subdomain models	Number of secondary or inferred cost items from R761/R762 classes per model class
Bridges and constructions	1–17
Construction details	1–5
Cabling systems	1–2
Earthworks	1
Landscaping	1
Lighting	3–4
Road	1
Road signs	1
Water and sewer	1–2

The sixth and final step was to import the classified quantities into the specification tool ISY Description. After importing the IFC file, aggregated quantities were listed according to their codes and titles. The tool had a built-in database containing all specification texts for standard works. Thereby a specification of work was automatically created.

Table 4 gives an overview of the mapping between the proprietary model classes including their respective property types, the R761/R762 main classes, and IFC 2 × 3 classes for the 195 automatically created cost items. IFC 2 × 3 classes do not cover all entities found in infrastructure projects. Related classes from the building domain—e.g., IfcBeam or IfcColumn—could be used for the subdomain models bridges and constructions and construction details, and to some extent for the subdomain models cabling systems and water and sewer, too—IfcFlowTerminal and IfcFlowSegment. However, for the subdomain models earthworks, landscape, lighting, road and road signs only IfcSlab or the generic IfcBuildingElementProxy were available.

**Table 4:** Mapping between the proprietary model classes, property types, the R761/R762 main classes and the IFC 2 × 3 classes for the 195 automatically created cost items.

V770 subdomain	Authoring tool	Proprietary model class	Property type 1	Property type 2	R761/R762 main class	IFC 2 × 3 classes
Bridges and constructions	Revit	Revit generic model	R761/R762 class set by Dynamo script	–	4, 5, 7, 8	IfcBeam; IfcBuildingElementProxy; IfcColumn; IfcCovering; IfcFlowSegment; IfcFooting; IfcMember; IfcPlate; IfcSlab; IfcWall; IfcReinforcingBar
Bridges and constructions	Tekla	Tekla generic object	R761/R762 class set by Grasshopper script	–	4, 5, 7, 8	IfcBeam; IfcBuildingElementProxy; IfcColumn; IfcFooting; IfcMember; IfcPlate; IfcRailing; IfcReinforcingBar; IfcSlab; IfcWall
Construction details	Revit	Revit generic model	R761/R762 class set by Dynamo script	–	7	IfcBeam; IfcColumn
Construction details	Novapoint	Shed	–	–	7	IfcSlab
Construction details	Civil 3D	NA	NA	NA	7	IfcBuildingElementProxy
Cabling systems	Novapoint	BaseShaft	object name (string)	–	4	IfcFlowTerminal
Cabling systems	Novapoint	ConduitPipe	layer name (string)	material/string	4	IfcFlowSegment
Cabling systems	Novapoint	EmbedmentLayer	layer name (string)	–	4	IfcSlab
Cabling systems	Novapoint	FillLayer	feature subtype (enum)	–	4	IfcSlab
Earthworks (roads)	Novapoint	Cut	material (enum)	–	2	IfcSlab
Earthworks (water and sewer/cabling systems)	Novapoint	Cut	–	–	4	IfcSlab
Landscape	Novapoint	DitchBottomSurface DitchSlopeSurface EmbankmentSlope	object name (string)	–	7	IfcSlab
Landscape	Novapoint	Kerb	layer name (string)	–	7	IfcSlab
Landscape	Civil 3D	NA	NA	NA	4, 6, 7	IfcBuildingElementProxy
Lighting	Civil 3D	NA	NA	NA	4, 7	IfcBuildingElementProxy
Road	Novapoint	StructureLayer	feature subtype (enum)	–	5	IfcSlab
Road	Novapoint	StructureLayer	layer name (string)	–	5	IfcSlab
Road	Novapoint	StructureLayer	object name (string)	–	7	IfcSlab
Road	Novapoint	SubgradeDeepBlasted	–	–	2	IfcSlab
Road	Novapoint	SubgradeSurface	layer name (string)	–	5	IfcSlab
Road signs	Novapoint	RoadSignMast	feature subtype (string)	–	7	IfcSlab
Water and sewer	Novapoint	BaseShaft	usage (string)	–	4	IfcFlowTerminal
Water and sewer	Novapoint	ConduitPipe	feature subtype (enum)	diameter/string	4	IfcFlowSegment
Water and sewer	Novapoint	FillLayer	feature subtype (enum)	–	4	IfcSlab
Water and sewer	Novapoint	SewerPipeline	material (enum)	diameter/string	4	IfcFlowSegment
Water and sewer	Novapoint	StormDrainLine	usage (enum)	diameter/string	4	IfcFlowSegment
Water and sewer	Novapoint	WaterPipeline	material (enum)	diameter/string	4	IfcFlowSegment

### Experiences with automated quantity take-off

The design team wanted to create a standardized and reusable system for automated quantity take-off in a road project. Such systems exist for buildings, but automated quantity take-off seems to rarely take place in road projects. This system was set up specifically for the investigated case but was created with potential reuse in other infrastructure projects in mind.

When it comes to standardization, the designers were partly successful. Random human errors caused by manually copy-pasting data and by different persons doing the same task differently were avoided. E.g., a terrain cut with the enumerated value "rock" as material was always automatically classified with the code 22.1, a water pipe with the enumerated value "ductile iron" as material and with a nominal diameter of 150 mm was always automatically classified with the code 43.222, or an embedment layer with the enumerated value "backfill" for the filling type was always classified with the code 44.12. However, classifying model classes was not always straightforward. There were two scenarios where this became apparent. Firstly, cost items involving processes—e.g., transport and mass disposal within the project site—were not modeled and could not be inferred, either. Secondly, some cost items priced with lump sums consisted of items with different attributes, for example, drawing pipes with different diameters. The quantification of these items required human attention.

When it comes to creating a reusable system, the designers were also partly successful. Fewer man-hours were needed to create the quantity take-off compared to a traditional drawing-based approach, especially at revisions. Only three steps—parsing, enriching, and importing—were necessary for revisions. This saved time in the investigated case since the project scope was downsized several times at different model maturity levels. Explicit time savings were not measured, but all actors agreed on significant time savings. However, the potential reuse within other infrastructure projects was limited. While most classifications for cost items with standard codes and primary quantities could be reused, classifications for cost items with non-standard codes and/or secondary or inferred quantities could only be reused to a limited extent. Put in other words, if there was a 1:1 mapping between the model class and the R761/R762 class—preferably with standard codes—the chances for reuse were much higher. This was especially apparent for the construction domain which had up to 17 secondary or inferred cost items from R761/R762 classes mapped to one model class.

## Discussion

So far, we have presented findings from action research on automated quantity take-off in a digitally advanced road project. We studied how quantity take-off is automated in the investigated case, and what the experiences from automated quantity take-off are.

### Automated quantity take-off in the investigated case

Four steps of the automated quantity take-off need to be discussed in detail. These are step 1, classification of proprietary model classes within authoring tools, step 3, parsing of the classified and enriched model classes to IFC 2 × 3, step 4, rule-based checking for data consistency, and step 5, additional enriching outside the authoring tools.

Step 1 is usually carried out in a specialized cost management tool. Model entities were classified either manually by using specific layer names and/or properties or semi-automated by using specific IFC classes and/or properties. Missing infrastructure-specific classes in IFC 2 × 3 limits the usability of these semi-automated workflows for the infrastructure domain. In the investigated case, this limitation was removed by hard coding model classes directly in the authoring tool. Thereby, newly created model entities belonging to previously mapped model classes were automatically classified. No human expert knowledge was necessary for this operation. Updated quantities were automatically transferred to their respective cost items in the specification tool.

However, hard coding was not always straightforward due to two reasons. Firstly, a generally accepted ontology for all physical assets (and thereby for digital assets) does not (yet) exist for the Norwegian infrastructure domain. It was therefore not always clear which assets the different model entities represented, especially in the geometry-based authoring tool Civil 3D where layer names or custom properties were the only sources of information. An ontology for all physical assets incorporating road-specific classes from IFC 4 × 3, Uniclass, Omniclass or CCI may help to circumvent this obstacle.

The second reason why hard coding was not always straightforward was that the R761/R762 classes represent work tasks and not physical assets. In some cases, up to 17 work tasks are necessary to create (and approve) a physical asset. E.g., the tasks 43.422 [Laying of water pipes of] Ductile iron [with a] diameter [of] 150 mm, 43.921 Flushing of pipes, 43.922 Cleansing of pipes, 43.923 Disinfection of pipes, and 43.924 Pressure testing of pipes are necessary to create and approve a water pipe. The tasks 71.14 [Establishing of a] Concrete sole, 71.15 [Laying of] Geotextile, and 71.16 Sorting, delivery and masonry are necessary to create a natural stone retaining wall. While several R761/R762 classes—primary, secondary, and/or inferred—could be assigned to one model entity by a script in Revit and Tekla, only one R761/R762 class could be assigned to one model entity in Novapoint. Secondary or inferred cost items required semantic enrichment in SimpleBIM (step 5). It was not always comprehensible for the designers which R761/R762 class was mapped directly in Novapoint, and which classes were added in SimpleBIM, especially for inferred cost items without a geometric entity. Furthermore, only one of these classes could be used as a classification in Solibri during step 4. Therefore, the validation process needed human attention and was only semi-automated.

Step 3, parsing of the classified and enriched model classes to IFC 2 × 3, was adapted to one specific Norwegian tool and one specific use case, namely creating a specification of work. The procedure was robust because it was independent of specific layer names or IFC classes. On one hand, this enabled automated quantity take-off for domains not yet covered by IFC 2 × 3 classes, namely the road and landscaping domain, because queries were based on proprietary classes. Furthermore, errors due to different authoring tools exporting similar model classes to different IFC classes were thereby avoided. On the other hand, it might limit the reuse of the quantities with other tools and other use cases. For example, calculating greenhouse gases based on the same quantities might not be straightforward due to different breakdown structures (e.g., all model entities from the road domain were exported as IfcSlab). Since the proprietary model classes (e.g., StructureLayer, SubgradeDeepBlasted or RoadSignMast for the road domain) and their respective properties were not exported, the breakdown structure could not be easily altered after parsing. Using IFC 4 × 3 classes could probably mitigate this obstacle.

### Experiences with automated quantity take-off

The design team wanted to create a standardized and reusable system for automated quantity take-off. This system was set up specifically for the investigated case but was created with potential reuse in other infrastructure projects in mind.

By hard coding model classes within authoring tools, the designers standardized the automated quantity take-off. On one hand, human errors caused by copy-pasting numbers manually or caused by a lack of experience were eliminated. On the other hand, systematic errors when mapping R761/R762 classes to model classes could still occur. While random human error could lead to varying quantities—either increasing or decreasing—after each iteration, a systematic (human) error always resulted in comparable quantities after each iteration.

However, the attempt to standardize the automated quantity take-off might have created a black box. Some designers seemed to blindly trust the results without being aware of the consequences of the hard coding. This was especially evident for mappings based on layer or object names (37% of all mappings in Novapoint). Some designers changed these names manually during revisions resulting in wrong or non-existing mappings between R761/R762 and model classes. However, the designers still trusted the correctness of the results. One reason for this blind trust might be the insufficient documentation of the mapping procedure. The document study revealed that the procedures did not stress the importance of a strict naming convention. Another reason could be wrong responsibilities for the hard coding. In the investigated case, three persons were responsible. For the construction domain, the construction discipline lead was responsible. The water and sewer designer was responsible for the water and sewer domain. The BIM manager was responsible for the road, electrical, and landscaping domains. The awareness was lowest for those domains mapped by the BIM manager. It does not seem appropriate to make the designers responsible for the hardcoding, because they are not always the ones responsible for the specification of work. To avoid black boxes, cost breakdown structures should not be hard coded in authoring tools.

Further, the significance of the 486 cost items needs to be discussed. The analysis of the specification of work showed that the R761/R762 main classes 4, 7, and 8 represented 72% of the 486 cost items with a share of respectively 19%, 33%, and 20% (see Table 2). However, the highest quantities were exported to the main classes 2, 4, 5, 6, and 7. This means that for the main classes 2, 5, and 6 few classes produce high quantities. Main class 1 contains work tasks for preparation and maintenance during the construction phase and the unit of measurement is mainly lump sum. No cost items in main class 1 were created automatically. To evaluate the significance of the cost items we asked the interviewed project participants. Based on their experience, they revealed that for projects with many constructions but without tunnels (like in the investigated case) the highest costs usually originate from the main classes 2, 5, and 8. In short, it seems most important to standardize the automated quantity take-off for the main classes 2, 5, and 8. Table 2 shows the share of automatically created cost items for all main classes. Main class 2 has a share of 5% (3 of 62 cost items), main class 5 has a share of 67% (6 of 9 cost items), and main class 8 has a share of 90% (88 of 98 cost items). More effort to standardize the automated quantity take-off for main class 2 seems necessary.

However, most quantities for manually created cost items in main class 2 need data processing due to temporary storage and transport on-site. This data processing seems difficult to achieve with standard tools. Two of the 59 manually created cost items for main class 2 were initially hard coded in the model. They were not used because manual operations were necessary to assign the correct R761/R762 class to the model entity. According to the designers, it took the same amount of time to create the quantities manually. They preferred a manual quantity take-off.

When it comes to the reusability of the hard coded mappings, the results are twofold. On one hand, it seems that the mappings worked well at revisions within the case investigated, especially when scripts were applied, or enumerated values were used as filters. Since all mappings in Revit and Tekla were based on scripts and 63% of all mappings in Novapoint were based on enumerated values the procedure appears to be well-suited for reuse. On the other hand, the transferability of the procedures to other projects seems highly questionable due to five reasons.

Firstly, using non-standard R761/R762 classes for cost items is very common. In the investigated case, 70% were non-standard classes that were specifically created for the project. The chances for reusing a project-specific, non-standard code seem rather low. Secondly, even standard codes can vary for the same model classes from project to project. For example, on-site materials can be used for creating the road embankment. In Norway, on-site materials are typically ordinary soil or blasted rock. If the predominant material is ordinary soil the fill layer is classified as 25.1. If the predominant material is rock, the fill layer is classified as 26.1. Thirdly, some of the R761/R762 classes seem abstract, especially to project members not familiar with them. Being an international design consulting company, COWI regularly distributes work to departments abroad. Unambiguous mappings are essential and the R761/R762 classes might confuse these foreign resources. Fourthly, the mappings might not work in early project phases because either the properties used for the filters or the model entity itself is not yet created. Applying the Level of Information Need (LOIN) may improve the reusability across project phases. Fifthly, a high number of R761/R762 classes per model class might reduce the reusability in other projects. Some model classes from the construction domain have up to 17 R761/R762 classes hard coded. The chances that the same 17 classes are used in other projects seem rather low. However, mappings for model classes with only one standard R761/R762 class appear to be well-suited for reuse in other projects.

### Parallels between findings and insights from the literature

Given the above, three major challenges stand out. Firstly, using ambiguous model classes, secondly, hard coding cost breakdown structures in authoring tools and thirdly reusing automated quantity take-off across project phases and use cases.

In the case under scrutiny, classified model entities were reused for automating quantity take-off. This seems to be the most common way described in the literature, too^19,20^. The literature reports further on two different approaches to classifying model entities, namely classifying proprietary model classes^21–23,26–29^ or classifying open model classes based on IFC^24,25^. In the investigated case, the two approaches were merged. Proprietary model classes were classified and serialized as IFC 2 × 3. The proprietary model classes acted as a starting point for queries. If no appropriate IFC 2 × 3 class from the building domain was available, generic IFC 2 × 3 classes were used. On one hand, this approach mitigated data exchange issues that^15^ identified as a challenge for automated quantity take-off. On the other hand, this approach amplified the black box described above. The third approach for automating the quantity take-off described in the literature was coding model entities by applying ontologies^16,33–38^. This approach was not utilized in the case under scrutiny. However, we realize the importance of unambiguous classes assembled in an ontology, especially for companies that distribute work internationally. Therefore, we recommend coding model entities by applying ontologies in future research.

In the literature, inconsistent information is often mentioned as a challenge for automated quantity take-off^10–14^. Therefore, human expert knowledge is necessary to classify model entities^16^. By hard coding proprietary model classes directly in the authoring tool, no human expert knowledge was necessary for the case under scrutiny. Newly created model entities belonging to previously mapped model classes were automatically classified. While this seemed to be a robust procedure at first, it might have created a black box. Therefore, we advise against hard coding of cost breakdown structures in authoring tools.

Another challenge for automated quantity take-off mentioned in the literature was geometric inaccuracy^5^. We did not assess the geometric accuracy of the models. This will be done as future research. Besides the geometric accuracy, the granularity of geometric information and alphanumeric information—the properties—needs to be discussed regarding the reusability of the automated quantity take-off presented. The mappings might not work in early design phases because either the properties used for the filters or the model entity itself is not yet created. Therefore, we suggest applying the Level of Information Need (LOIN) to improve reusability across project phases and use cases. Design consulting companies can apply LOIN regardless of whether the client—like the NPRA—requires employer-specific standards. Thereby, design consulting companies can establish an ISO-compliant system but satisfy employer-specific requirements at the same time. This is especially important for an international design consulting company like COWI.

## Conclusion

Automated quantity take-off takes place in building projects and other types of engineering projects, but not in road projects. This paper investigates automated quantity take-off in road projects after addressing how quantity take-off is automated in a real-life road project and the experiences from this automation. In the investigated case, automated quantity take-off was standardized for 40% of all 486 cost items. The automated workflow was possible to achieve with commercial software and a publicly available cost breakdown structure (R761/R762). The automated workflow was based on standardized data. Data were standardized by hard coding proprietary model classes according to the cost breakdown structure and parsing them as IFC 2 × 3 classes.

The experience was that the automated quantity take-off was reproducible at revisions within the case investigated. However, the transferability of the procedures to other projects—and other use cases—seems highly questionable. This is mainly due to the nature of the applied cost breakdown structure (R761/R762) with wide use of non-standard codes but to a minor degree also due to a lack of modelled elements in early project phases.

The paper documents digital ways of working in infrastructure projects that have been suggested in the literature, but for which there is limited empirical evidence available. Three practical suggestions for improving the reusability of automated quantity take-off in road projects can be derived from the findings. Firstly, to standardize the exported data structure, we recommend the use of unambiguous classes assembled in an ontology. Generally accepted concepts in natural language, e.g., "wearing course", "pipe", or "cable", should be used for model classes instead of proprietary model classes or layer names. Properties used as filters—e.g., material, dimension, or usage—should be exported in a standardized form. Secondly, to avoid black boxes, cost breakdown structures should not be hard coded in authoring tools. Thirdly, to improve reusability across project phases and use cases the Level of Information Need (LOIN) should be applied. For each project phase the required domains, entities, properties, and dimensionality should be defined. E.g., for early project phases only lengths of roads, bridges and tunnels are necessary, while several units of measurement and properties about materials, dimensions, or usage are necessary for detailed design.

Although the presented findings stem from only one Norwegian road project, the results should be relevant for researchers and practitioners from other nations. The action research approach helped to identify three practical experiences mentioned above that improve reusability. Firstly, unambiguous classes assembled in an ontology should be used. The ontology should incorporate road-specific classes introduced in IFC 4 × 3, Uniclass, Omniclass or CCI. Secondly, hard coding of cost breakdown structures in authoring tools should be avoided. Thirdly, the Level of Information Need (LOIN) should be applied to improve reusability across project phases and use cases. A LOIN definition is required per use case, e.g., cost estimation, calculation of greenhouse gases, assessment of environmental impacts, or scheduling.

Future research should build on the three suggested points for improvement. Firstly, unambiguous classes assembled in an ontology should be used for standardizing the exported data structure. Secondly, cost breakdown structures should not be hard coded in authoring tools. Thirdly, the Level of Information Need (LOIN) should be implemented in this work.

## Data Availability

The datasets generated during the current study are available on reasonable request. Correspondence and requests for materials should be addressed to D.F.
